# The Retrogression of Cancer

**Published:** 1896-12-12

**Authors:** 


### Editor's Letter-Box.

### THE RETROGRESSION OF CANCER.

Dr. George Stoker writes: I was much interested by your remarks on "Retrogression in a Malignant Case" that was reported at the Clinical Society. I have at this moment in the Oxygen Home a case in which malignant growth is retrograding rapidly under treatment, and that a very simple and scientific treatment, *i.e.*, oxidation. I feel sure you are right in supposing that some chemical action is at work which holds the balance between the favourable and unfavourable development of cell life, and that chemical action I believe to be oxidation. It is not only possible but extremely likely that it is a want of, a depreciation of, this process that leads to malignant and other new growths; my case would certainly point this way.

